# Construction of Luminogen Exhibiting Multicolored Emission Switching through Combination of Twisted Conjugation Core and Donor-Acceptor Units

**DOI:** 10.3390/molecules22122222

**Published:** 2017-12-14

**Authors:** Haiyan Tian, Xi Tang, Yong Qiang Dong

**Affiliations:** Beijing Key Laboratory of Energy Conversion and Storage Materials, Department of Chemistry, Beijing Normal University, Beijing 100875, China; 201431150053@mail.bnu.edu.cn (H.T.); hptangxi@163.com (X.T.)

**Keywords:** aggregation-induced emission, multicolor emission switching, mechanochromic fluorescence, optical recording

## Abstract

Stimuli responsive luminescent materials, especially those exhibiting multicolor emission switching, have potential application in sensor, optical recording, security ink, and anti-counterfeit label. Through combination of twisted conjugation core and donor and acceptor units, a luminogen (2-(*bis*(4-(carbazol-9-yl)phenyl)methylene)malononitrile (**1**) was synthesized. Luminogen **1** can form three kinds of crystals emitting green (**1GC**, λ_em_ = 506 nm, Φ_F_ = 19.8%), yellow-green (**1YC**, λ_em_ = 537 nm, Φ_F_ = 17.8%), and orange (**1OC**, λ_em_ = 585 nm, Φ_F_ = 30.0%) light upon 365 nm UV illumination. The emission of amorphous solid of **1** (**1Am**) overlaps with that of **1OC** (λ_em_ = 585 nm), with quantum yield of 13.9%, which is seldom reported. Emission of **1** can be switched among green, yellow-green, and orange through morphology modulation upon exposure to thermal, solvent vapor, or mechanical stimuli. Finally, its potential application in optical recording was also investigated.

## 1. Introduction

Stimuli responsive luminescent materials have attracted much attention in recent years due to their potential application in optical storage [1], optoelectronic devices [2,3,4], security papers [5], and sensor [6,7,8,9,10,11,12,13,14,15]. The emission colors and intensities of these materials can be tuned by external stimuli, such as heat [16,17,18], solvent vapor [19,20,21], and mechanical perturbation (including grinding, shearing, smashing, or stretching) [22,23,24,25,26]. However, their practical applications are limited because emissions of many luminogens are weakened or totally quenched in the solid state, which is known as aggregation-caused quenching (ACQ) [27,28].

In 2001, Tang found that a series of propeller-like luminogens were nearly nonemissive in solution but emitted intensely upon aggregation, thus, Tang coined this phenomenon as aggregation-induced emission (AIE) [29,30]. The phenomenon was mainly ascribed to the restriction of intramolecular motions (RIM) process [31]. In 2005, Tang and coworkers found that crystals of some AIE luminogens exhibited more intense emission than their amorphous solid, and they coined this phenomenon as crystallization induced emission enhancement (CIEE) [32,33]. The molecules of AIE luminogens pack loosely and can easily form varied morphologies due to their flexible conformations. The loose packing patterns of those CIEE active luminogens also facilitate the reversible transformation between different morphologies. Thus, the emission of many CIEE active luminogens can be switched between different states through morphological modulation [34,35,36].

Although many stimuli responsive luminogens that are based on CIEE activity have been developed [37,38,39,40,41,42,43,44], emission of most of them can only be switched between two states through morphology tuning between amorphous and crystalline states [23,25,41,44,45,46]. Luminogens exhibiting multicolored switching are rather rare due to the lack of design strategy, in spite of their potential for improving anti-fake complexity and density of optical storage.

D-π-A type materials usually exhibit unique fluorescence properties due to their intramolecular charge transfer (ICT) transitions [47,48,49]. However, ACQ often takes place in the condensed phases for many D-π-A luminogens, limiting their real-world applications. In this paper, we constructed a symmetric compound 2-(*bis*(4-(9H-carbazol-9-yl)phenyl)methylene)malononitrile (**1**), with electron donor (carbazole) and acceptor (two cyano groups). Luminogen **1** is AIE and CIEE active and can form four morphologies with varied emissions and efficiencies. In particular, luminogen **1** can be switched among three different crystalline forms with green, yellow-green, and orange emission, which is rarely reported. Luminogen **1** exhibits mechanochromic luminescence and its potential application in optical recording was investigated.

## 2. Results and Discussion

### 2.1. Synthsis of Compound ***1***

**Scheme 1:**
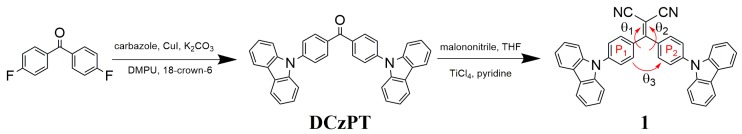
Synthesis routes to compound **1.**

Compound **1** was prepared according to the synthetic procedures shown in Scheme 1, giving a yield of 86%. The structure of **1** was characterized by ^1^H-NMR, ^13^C-NMR, and HRMS spectroscopies (Figures S1–S4). The detailed synthetic procedures can be found in the Materials and Methods of this paper. Thermal gravimetric analysis (TGA) showed that compound **1** possessed good thermal stability, losing 5% of its weight under nitrogen at about 411 °C (Figure S5).

### 2.2. The UV-Vis Absorption and Aggregation-Induced Emission (AIE)

The UV-Vis absorption of **1** in dichloromethane (DCM) was showed in Figure S6. The maximum absorption of **1** locates at 414 nm and the molar absorption coefficient ε is 15,707 L/(mol·cm), which is quite high among D-π-A compounds. Compound **1** is nearly nonemissive when it is dissolved in acetonitrile, while the emission is turned on upon addition of large amount of water to the solution. Compound **1** is hydrophobic and forms aggregates upon addition of large amount of water. That is, compound **1** is AIE active (Figure S7).

### 2.3. Emission of the Three Crystals and the Amorphous Solid

**Scheme 2:**
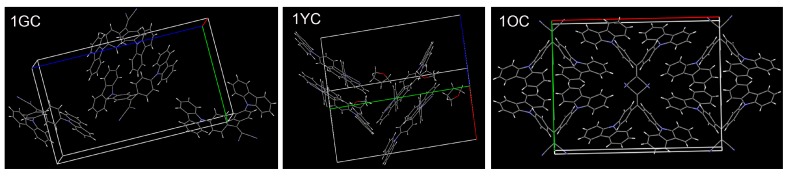
Schematic illustration of the three single crystals of **1GC**, **1YC,** and **1OC**.

**Figure 1:**
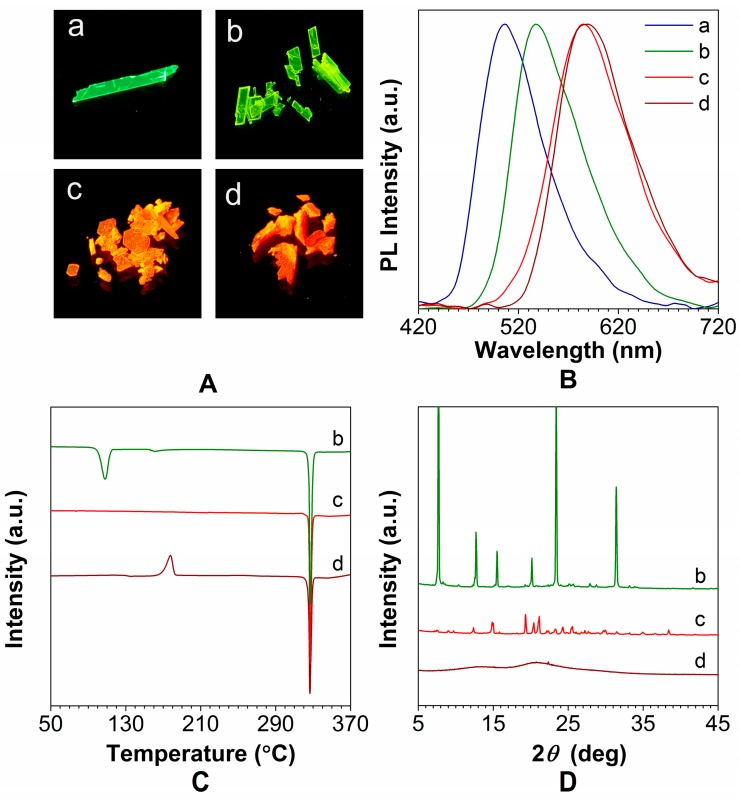
(**A**) Photos of compound **1** in different states: (**a**) **1GC**, (**b**) **1YC**, (**c**) **1OC** and (**d**) **1Am**; (**B**) Normalized PL spectra of samples (**a**–**d**), excitation wavelength: 370 nm; (**C**) differential scanning calorimetry (DSC) curves and (**D**) Powder X-Ray diffraction (PXRD) patterns of samples (**b**–**d**).

The photophysical properties of luminogen **1** in the solid state were further studied because luminogens are generally used in solid state in their real applications. We obtained three kinds of single crystals **1GC** (λ_em_ = 506 nm, Φ_F_ = 19.8%, Figure 1A (a)), **1YC** (λ_em_ = 537 nm, Φ_F_ = 17.8%, Figure 1A (b)) and **1OC** (λ_em_ = 585 nm, Φ_F_ = 30.0%, Figure 1A (c)) through slow evaporation the mixture of DCM/*n*-hexane (volume, 3:1), THF/*n*-hexane (volume, 2:1) and EA/*n*-hexane (volume, 1:1), respectively, at room temperature under the rigorous exclusion of light. **1GC** and **1OC** belong to orthorhombic system without any solvent molecule in the lattice; **1YC** belongs to monoclinic system with tetrahydrofuran (THF) molecules in crystal lattice, and the molecular molar ratio of **1** versus THF is 1:1, which was also confirmed by ^1^H-NMR (see Figure S1).The schematic illustration of three crystals is shown in Scheme 2.

In addition to the single crystal structure and quantum efficiency, photoluminescence (PL) spectra (Figure 1B), differential scanning calorimetry (DSC) curves (Figure 1C), and Powder X-Ray diffraction (PXRD) patterns (Figure 1D) of **1OC** and **1YC** also reveal that they are different crystalline phases. Unfortunately, we cannot obtain enough **1GC** for the measurements of DSC and PXRD.

Many AIE luminogens are CIEE active, so we prepared the amorphous sample of luminogen **1** (**1Am**, λ_em_ = 585 nm, Φ_F_ = 13.9%, Figure 1A (d)) by quenching its melt with liquid nitrogen, and the amorphous essence of **1Am** was verified by the absence of diffraction peaks in its PXRD pattern (Figure 1D, line d). Exothermic peak at 177 °C in the DSC curve of **1Am** (Figure 1C, line d) indicates the crystallization transition. Thus, luminogen **1** exhibits four morphologies, depending on different molecular packing modes in the solid state. The optical properties such as florescence lifetime and quantum yield of four aggregate states were summarized in Table 1.

The availability of several single crystals based on one luminogen provides the possibility to study the effect of molecular conformation and packing pattern on the photophysical properties of the luminogen, ruling out the effect of chemical structure. The extraordinary twisted molecular conformation of **1** in crystals rule out the strong intermolecular interactions, such as π-π stacking [22,23] or H/J aggregates [50,51]. Thus, the difference of emission colors among the three crystals may be attributed to the difference of molecular conformation. Obviously, the dihedral angles of **1OC** are smaller than that of **1GC**, suggesting better coplanarity and greater conjugation of **1OC**, which is responsible for red-shifted emission of **1OC**. The dihedral angles of **1YC** are smallest among three crystals, however, its emission color falls in between **1GC** and **1OC** (Table S4). This abnormal phenomenon may be caused by the THF molecules in **1YC [52]**. **1Am** exhibits similar emission to **1OC**, which may be caused by the similar molecular conformation in different molecular packing modes.

The crystals of **1** show higher quantum yields than **1Am,** as molecules in crystalline states pack more tightly than those in **1Am**, which may further hinder the rotation of phenyl rings and block the nonradiative transmission pathway. **1OC** exhibits strongest emission among three crystals, closely followed by **1GC**, finally **1YC**, which is in good agreement with the number of interactions existing in crystals: four C≡N···H and thirty six C–H···π interactions in **1OC**, two C≡N···H and twenty two C–H···π interactions in **1GC** and two C–H···O, nine C≡N–H and twelve C–H···π interactions in **1YC** (Figures S8–S10 and Tables S5–S7).

### 2.4. Tuning the Emission of ***1*** in the Solid State

Emission of many CIEE active luminogens can be switched between two states through morphology tuning between amorphous and crystalline states. However, examples of multicolored emission switching are rather rare. If we could modulate the molecular packing patterns of **1**, then we may switch its emission in the solid state among multiple colors [53].

**Figure 2:**
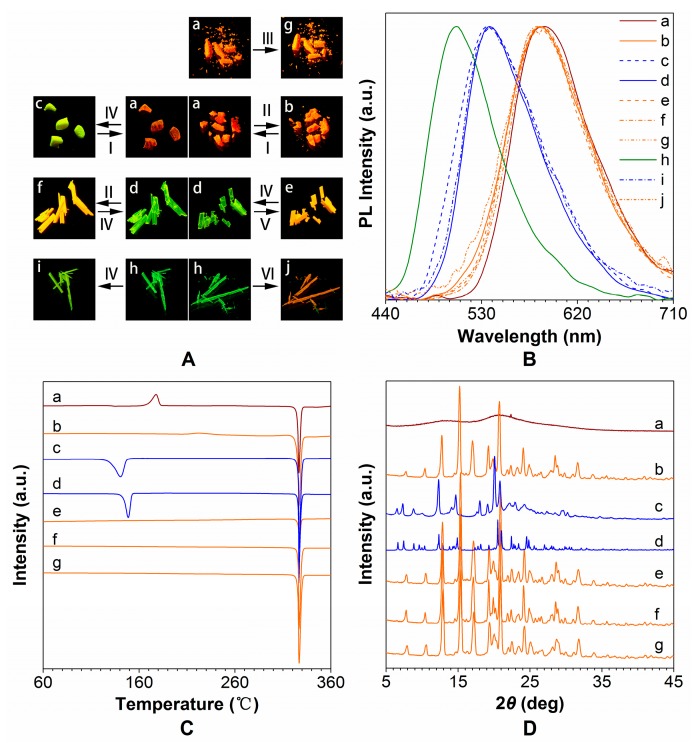
(**A**) Photographs of samples (**a**–**j**) under the 365 UV illumination: (**a**) **1Am**, (**b**) **1Am** fumed with ethyl acetate (EA), (**c**) **1Am** fumed with tetrahydrofuran (THF), (**d**) **1YC**, (**e**) **1YC** annealed at 140 °C, (**f**) **1YC** fumed with EA, (**g**) **1Am** heated at 200 °C, (**h**) **1GC**, (**i**) **1GC** fumed with THF, (**j**) **1GC** fumed with DCM; (**B**) Normalized PL spectra of samples (**a**–**j**), excitation wavelength: 370 nm; (**C**) DSC curves and (**D**) PXRD patterns of samples (**a**–**g**). Conditions: I: heating to melt and quickly cooled by liquid nitrogen; II: fuming with EA; III: heating at 200 °C; IV: fuming with THF; V: heating at 140 °C; and, VI: fuming with DCM.

Normally, an amorphous solid will crystallize upon heating or exposure to solvent vapor. Inspired by the exothermic peak at 177 °C in the DSC curve of **1Am**, we heated **1Am** at 200 °C. However, **1Am** exhibited nearly no change in emission color (Figure 2A, (a) to (g)). Although **1Am** and **1OC** are both orange emissive, the transformation of **1Am** to **1OC** upon heating was verified by the overlapping of the DSC curves and PXRD patterns (lines a and g in Figure 2C,D). In addition to thermal treatment, we exposed **1Am** to solvent vapor, finding that **1Am** transformed to **1OC** upon exposure to ethyl acetate (EA) (Figure 2A, (a) to (b)), while to **1YC** upon exposure to THF (Figure 2A, (a) to (c)). The transformation was further verified by PL spectra, DSC curves, and PXRD patterns (lines b and c in Figure 2B–D). Therefore, the weak orange emissive **1Am** can be converted to bright orange **1OC** upon heating at 200 °C or fuming with EA vapor, while to yellow-green **1YC** when fumed with THF. We could not obtain **1GC** through solvent fuming though we have tried many solvents. **1Am** can be re-obtained by quenching the melt of **1** with liquid nitrogen. Thus, we can switch the emission of **1** reversibly between weak orange and bright orange, or between weak orange and yellow-green.

In addition to switching **1** between amorphous and crystalline states, we tried to modulate luminogen **1** among multiple crystalline states. The endothermic peak at around 128 °C in the DSC curve of **1YC** (line d in Figure 2C) indicates a crystal to crystal phase transition, so we heated the **1YC** at 140 °C and found that the emission of **1YC** changed from yellow-green to orange (Figure 2A, (d) to (e)). The PL spectrum, DSC curve, and PXRD pattern of the heated **1YC** fit well with those of **1OC**, indicating that **1YC** had transformed to **1OC** upon heating at 140 °C. However, no thermal effect was observed in the DSC curve of **1OC** before melt, therefore, **1OC** could not convert back to **1YC** by thermal treatment. In addition to the thermal treatment, we tried to achieve the transformation among different crystalline phases of **1** through solvent fuming. Fortunately, **1YC** turned to **1OC** upon fuming with EA vapor (Figure 2A, (d) to (g)), while **1OC** can transform back to **1YC** upon exposure to THF vapor (Figure 2A, (e) to (d) and (f) to (d)). This transformation was easily distinguished by the naked eye and verified by PL spectra, DSC curves, and PXRD patterns (Figure 2B–D).

As aforementioned, we obtained **1YC** in THF/*n*-hexane and found THF molecules existing in the **1YC**, however, **1OC** had no solvent molecule in the lattice. So, we guessed it was the loss of THF molecules that led to the transformation from **1YC** to **1OC**. In order to verify our assumption, we heated **1YC** at 90 °C in vacuum for some time and found the yellow-green crystals changed to orange. This change was recorded by ^1^H-NMR (Figures S1 and S2). We heated the **1YC** at 90 °C so that the THF molecules were removed without changing crystal phase (the crystal transformation temperature is about 128 °C). Therefore, the reason of **1YC** to **1OC** is indeed the loss of THF molecules; when the THF molecules accessed into **1OC** again, **1YC** was obtained again. Therefore, we can draw the conclusion that the THF molecules can change molecular conformation and packing mode of **1YC** and **1OC [54]**. Inspired by above phenomenon, we also obtained **1YC** by fuming **1GC** with THF vapor (Figure 2A, (h) to (i)), while we got **1OC** by fuming **1GC** with DCM (Figure 2A, (h) to (j)), which was verified by the PL spectra (Figure 2B). Unfortunately, we could not measure the DSC curves and PXRD patterns about the transformation process of **1GC** because of few **1GC** we obtained. Thus, the emission of luminogen **1** can be switched reversibly between orange (**1OC**) and yellow-green (**1YC**) by repeating annealing at 140 °C (or fuming with EA vapor) and exposure to THF vapor. The reversible transformation process can be repeated many times due to the physical nature of the process (Figures S12–S14). Besides, we achieve the switching of **1GC** to **1OC** by fuming with DCM or **1GC** to **1YC** upon exposure to THF vapor. Thus, we can switch the emission of a single compound among three crystals in the solid state, which is rarely reported.

### 2.5. Mechanochromic Fluorescence

Mechanical stimuli normally amorphizes crystals of luminogens, and the amorphous solid will crystallize again upon heating or fuming with solvent vapor, thus, luminogen **1** may exhibit mechanochromic luminescence and its emission may be reversibly switched between different colors by thermal, mechanical, and solvent stimuli.

**Figure 3:**
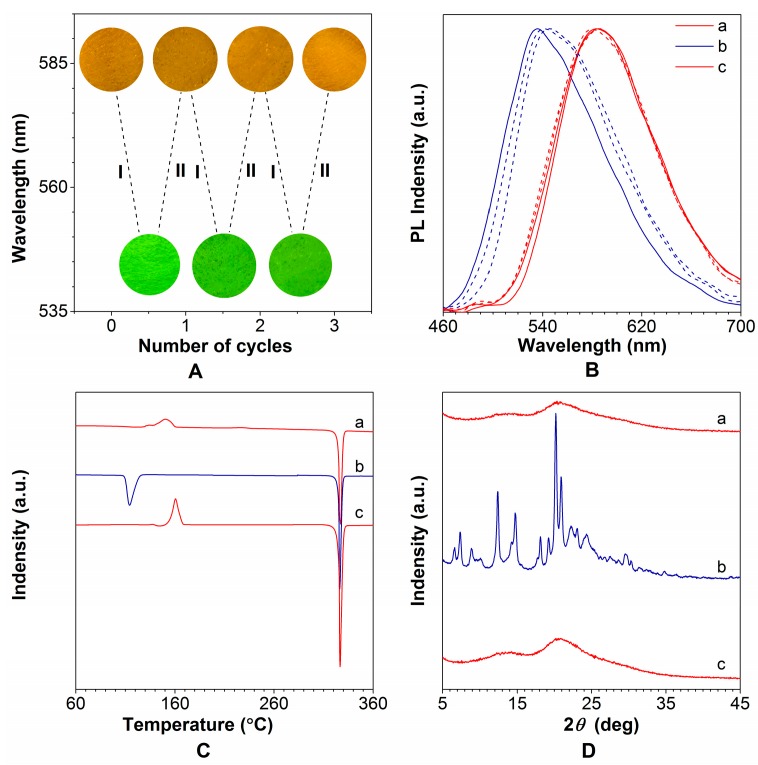
(**A**) Switching the fluorescence and photograph of **1** by repeated grinding (II) and fuming with THF (I) in the agate mortar; (**B**) Normalized PL spectra of ground powder and fumed solid of **1** in the three repeating cycles, the blue dotted lines: ground powder fumed with THF and the red dotted lines: ground powder, excitation wavelength: 370 nm; (**C**) DSC curves and (**D**) PXRD patterns in the first cycle. (**a**) ground powder, (**b**) sample (a) fumed with THF, (**c**) sample (b) ground again.

**Figure 4:**
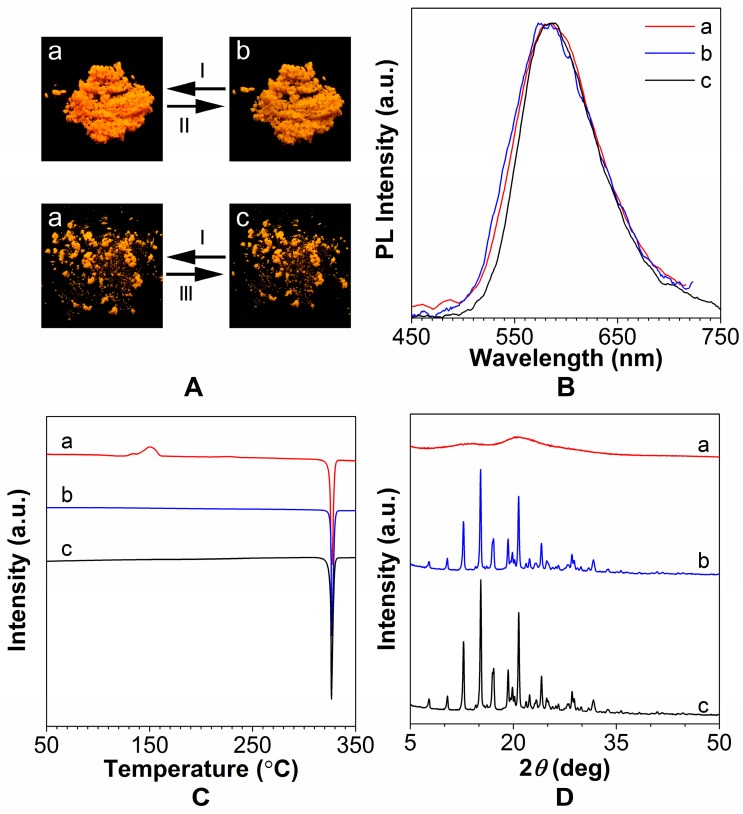
(**A**) Photographs of (**a**) ground solid from **1OC**, (**b**) sample (a) fumed with EA and (**c**) sample (a) annealed at 185 °C; (**B**) Normalized PL spectra, (**C**) DSC curves, and (**D**) PXRD patterns of samples (**a**–**c**). Conditions: I: grinding; II: fuming with EA; III: heating at 185 °C.

The yellow-green emissive **1YC** turned to orange upon grinding and the PL spectrum of the ground powder overlapped with that of **1Am**. In addition, both the exothermal peak in the DSC curve and absence of diffraction peaks in PXRD pattern of the ground powder from **1YC** suggested that **1YC** had been amorphized by grinding (Figure 3). As aforementioned, **1Am** can transform to **1YC** upon exposure to THF vapor; thus, we fumed the ground powder of **1** with THF. As we expected, the emission of the ground powder turned to yellow-green (Figure 3A), indicating that the ground powder turned to **1YC**, which was verified by the PL spectrum, DSC curve, and PXRD pattern (line b in Figure 3B–D). Emission color and the PL spectrum of the ground powder did not change upon exposure to EA vapor or annealing at 185 °C, which was similar to **1Am** (Figure 4A,B). However, the DSC curves and PXRD patterns of the ground powder after fuming or heating fit well with those of **1OC** (Figure 4C,D). Thus, luminogen **1** exhibits mechanochromic luminescence, and the emission of **1** can be reversibly switched between orange and yellow-green by alternate grinding and THF fuming process. The complexity of the transformation of the ground powder upon exposure to different external stimuli may afford its potential application in security ink and optical recording.

### 2.6. Application in Optical Recording

**Figure 5:**
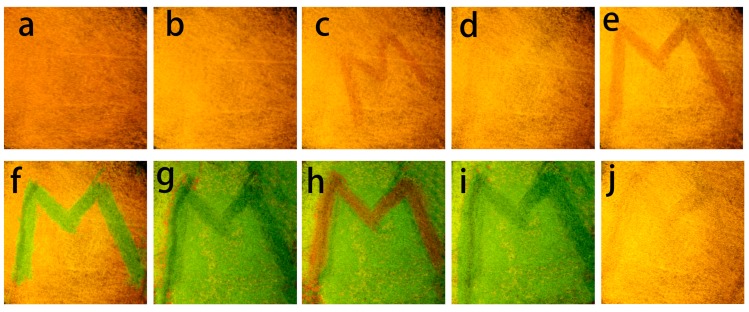
Procedures of writing and erasing process of using the weighing paper coated with **1**, luminescent images were taken under 365 nm UV illumination: (**a**) **1** ground on weighing paper; (**b**) a annealed at 185 °C; (**c**) wrote “M” in b; (**d**) c fumed with EA; (**e**) rewrote “M” in d; (**f**) e fumed with THF for 30 min; (**g**) f fumed with THF for 2.5 h; (**h**) wrote “M” in g; (**i**) h fumed with THF for 10 min, (**j**) i annealed at 140 °C for 10 min.

The multicolored mechanochromic fluorescence of **1** prompts us to investigate its potential application as an optical recording material. Luminogen **1** was ground on one piece of weighing paper, and then the weak orange paper (Figure 5a) turned to bright orange (Figure 5b) after annealing at 185 °C. Then, we wrote “M” on the “paper”, a weak orange “M” appeared on the bright orange background due to the amorphization of **1OC** in the sheared handwriting area (Figure 5c). The letter “M” disappeared when the “paper” was fumed with EA (Figure 5d), due to the transformation of **1** in the letter area from **1Am** to **1OC**. We rewrote “M” on the “paper”, and then a weak orange “M” appeared again on the bright orange background (Figure 5e). When the “paper” was exposed upon THF vapor for 30 min, a yellow-green “M” appeared on the orange background due to the transformation of **1** in the area of “M” from **1Am** to **1YC** (Figure 5f). However, the orange background kept unchanged in the process because the transformation of **1OC** to **1YC** is much slower than the transformation from amorphous solid to crystal. The whole “paper” turned to yellow-green when it was fumed with THF vapor for another 2.5 h (Figure 5g). We rewrote “M” on the “paper”, an orange “M” appeared on the yellow-green background (Figure 5h) and the “M” disappeared when the “paper” was fumed with THF vapor for 10 min (Figure 5i). Finally, the yellow-green “paper” returned to bright orange when annealed at 140 °C for 1 h (Figure 5j). Compound **1** exhibits different response to different stimuli and the emission changes can be distinguished by the naked eyes, suggesting its potential application in anti-fake.

## 3. Materials and Methods

### 3.1. Materials and Instruments

All of the original material were commercially available and used without further purification. ^1^H-NMR and ^13^C-NMR spectra were measured on a Bruker AV III 400 MHz Spectrometer (Bruker, Karlsruhe, Germany). Mass spectrum was recorded with an AB SCIEX Triple Top 5600 mass spectrometer (AB SCIEX, Framingham, MA, USA), equipped with a dual sprayer orthogonal electrospray source (Lock Spray). Thermal gravimeter analysis (TGA) was performed using a METTLER TGA/DSC instrument (Mettler, Greifensee, Switzerland) at a scanning rate of 10 K/min. UV-Vis absorption spectra were recorded on a Shimadzu UV-2450 recording spectrophotometer (Shimadzu, Kyoto, Japan). The fluorescence spectra were recorded on a VARIAN Cary Eclipse fluorescence spectrophotometer (Varian, Melbourne, Australia). Fluorescence quantum yield was recorded on Hamamatsu Quantaurus-QY C9220-02 (Quantaurus-QY, Hamamatsu, Japan) at room temperature with a calibrated integrating sphere system. Differential scanning calorimetry (DSC) curves were performed on a METTLER DSC 1 instrument (Mettler) at a heating rate of 10 K/min from room temperature to 360 °C under nitrogen atmosphere. Powder X-Ray diffraction (PXRD) patterns were performed on a BDX-3000 (Beijing university electronic instrument factory, Beijing, China) with Cu Kα radiation (λ = 1.5418 Ǻ) at 25 °C (scan range: 4.5–50°). Single crystals data was collected on a Bruker APEX-II CCD diffractometer (Bruker), using graphite monochromatic Mo Kα radiation (λ = 0.71073 Ǻ). All of the photographs were taken by PENTAX K-5 digital camera (PENTAX, Kyoto, Japan) and all photos in the same Figure were same exposure time. The thermal annealing process was carried out in oven. The amorphous powder of luminogens was prepared by heating the luminogens to melt with a heating gun and quenching the melt with liquid nitrogen.

### 3.2. Synthesis of Compound ***1***

*Synthesis of bis(4-(9H-carbazol-9-yl)phenyl)methanone* (**DCzPT**). Under an argon atmosphere, *bis*(4-fluorophenyl)methanone (4.45 g, 20 mmol), carbazole (17.3 g, 103 mmol), potassium carbonate (5.02 g, 36 mmol), CuI (0.27 g, 14 mmol) and 18-crown-6 (0.17 g, 0.6 mmol) were added in a 1000 mL two-neck flask. The mixture was added into 1 mL DMPU and then heated up to 170 °C for 10 h. After cooling to room temperature, the mixture was extracted with vast DCM and saturated NaCl solution until the water layer was neutral. The organic layer was dried over MgSO_4_ and was concentrated by a rotary evaporator. The crude product was separated by silica gel column chromatography (eluent: TCM/*n*-hexane). Finally faint yellow-green solid was obtained in a yield of 68%. ^1^H-NMR (400 MHz, DMSO-*d*_6_), δ (TMS, ppm): 8.29 (d, 4H), 8.18 (d, 4H), 7.92 (d, 4H), 7.60 (d, 4H), 7.50 (t, 4H), 7.35 (t, 4H). ^13^C-NMR (100 MHz, DMSO-*d*_6_), δ (TMS, ppm): 199.07, 146.06, 144.86, 140.63, 137.00, 131.70, 131.55, 128.41, 125.88, 115.09.

*Synthesis of 2-(bis(4-(9H-carbazol-9-yl)phenyl)methylene)malononitrile* (**1**). **DCzPT** (5.13 g, 10 mmol) and malononitrile (1.43 g, 23 mmol) were added in a 250 mL two-neck flask. The mixtures was dissolved in 60 mL anhydrous chloroform under Ar atmosphere. Then, 4.4 mL TiCl_4_ was slowly added into the flask at 0 °C. 5 mL pyridine was dropwise added to the reaction mixture and stirred for 2 h at room temperature. The reaction was quenched with excess distilled water and then extracted with vast TCM and saturated NaCl solution. The organic layer was dried over MgSO_4_ and concentrated by a rotary evaporator. The crude product was purified by recrystallization. Yellow-green solid power was obtained in 86.0% yield. The purified product was characterized using spectra spectroscopic methods. ^1^H-NMR (400 MHz, CDCl_3_), δ (TMS, ppm): 8.16 (d, 4H), 7.83 (s, 8H), 7.58 (d, 4H), 7.46 (td, 4H), 7.35 (td, 4H). ^13^C-NMR (100MHz, CDCl_3_), δ (TMS, ppm): 172.31, 142.29, 139.95, 133.86, 132.41, 126.61, 126.37, 124.09, 121.02, 120.58, 114.00, 109.81, 81.60. HRMS (ESI) *m*/*z*: [M+] calcd. for C_40_H_24_N_4_, 561.2035; found, 560.9629. Crystal Data for **1GC** (C_40_H_24_N_4_ ): orthorhombic, space group P2(1)2(1)2(1), a = 7.907(2) Å, b = 14.819(4) Å, c = 26.414(7) Å, β = 90.0°, V = 3095.1(13) Å^3^, Z = 4, T = 100.0(2) K, λ(Mo Kα) =λ = 0.71073 Ǻ , Dcalc = 1.203 g/cm^3^, 17606 reflections measured (4.132° ≤ 2Θ ≤ 50.500°), 5609 unique (Rint = 0.0522) which were used in all calculations. The final R1 was 0.0525 (I > 2σ(I)) and wR2 was 0.1259. Crystal Data for **1YC** (C_40_H_24_N_4_ ): monoclinic, space group P2(1), a = 10.3034(16) Å, b = 23.763(4) Å, c = 13.737(2) Å, β = 104.447(3)°, V = 3257.0(8) Å^3^, Z = 4, T = 100.0(2) K, λ(Mo Kα) =λ = 0.71073 Ǻ, Dcalc = 1.290 g/cm^3^, 18,602 reflections measured (3.428° ≤ 2Θ ≤ 50.498°), 11,381 unique (Rint = 0.0358) which were used in all calculations. The final R1 was 0.0533 (I > 2σ(I)) and wR2 was 0.1270. Crystal Data for **1OC** (C_40_H_24_N_4_ ): orthorhombic, space group Pbcn, a = 22.459(5) Å, b = 16.839(4) Å, c = 7.8968(18) Å, β = 90°, V = 2986.5(12) Å^3^, Z = 4, T = 100.0(2) K, λ(Mo Kα) =λ = 0.71073 Ǻ , Dcalc = 1.247 g/cm^3^, 18,984 reflections measured (3.626° ≤ 2Θ ≤ 55.044°), 3436 unique (Rint = 0.0441), which were used in all of the calculations. The final R1 was 0.0407 (I > 2σ(I)) and wR2 was 0.0875.

CCDC 1583901 for **1GC**, 1583900 for **1YC** and 1583902 for **1OC** contain the supplementary crystallographic data for this paper. These data can be obtained free of charge from The Cambridge Crystallographic Data Centre via http://www.ccdc.cam.ac.uk/conts/retrieving.html.

## 4. Conclusions

Through introduction of donor and acceptor units to twisted conjugation core, a luminogen (**1**) exhibiting morphology dependent emission and multicolored emission switching was obtained. Luminogen **1** can form green, yellow-green, and orange emissive crystals, as well as orange emissive amorphous solid. The twisted conformation affords loose packing patterns and facilitates emissive switching through morphology tuning. Luminogen **1** exhibits mechanochromic luminescence property, which affords its potential application in security inks and optical recording. More luminogens exhibiting morphology dependent emission and mechanochromic luminescence may be obtained through the combination of D-A units with twisted conformation.

## Figures and Tables

**Table 1:** Optical properties of luminogen **1** in the various aggregate states.

Samples	^a^ λ_em_ (nm)	^b^ Φ_F_ (%)	^c^ ⟨τ⟩ (ns)
**1GC**	506	19.8	6.59
**1YC**	537	17.8	18.66
**1OC**	585	30.0	23.23
**1Am**	585	13.9	17.76

^a^ λ_em_ = Emission maximum. ^b^ Φ_F_ = Fluorescence quantum yield determined using a calibrated integrating sphere, excited at 370 nm. ^c^ The mean lifetime ⟨τ⟩ was calculated according to ⟨τ⟩ = (*A*_1_τ_1_ *+ A*_2_τ_2_)/(*A*_1_ *+ A*_2_).

## Supplementary Materials

The supplementary materials are available online.
