# Supplementary material for: Construction of Luminogen Exhibiting Multicolored Emission Switching through Combination of Twisted Conjugation Core and Donor-Acceptor Units
Source: Molecules. 2017 Dec 14;22(12):2222. doi: 10.3390/molecules22122222 (PMC6149696; doi:10.3390/molecules22122222)
Supplement: Supplementary file 1 [file molecules-22-02222-s001.pdf]

Electronic supplementary information (ESI) of the manuscript entitled
"Construction of luminogen exhibiting multicolored emission switching through combination of twisted conjugation core and donor-acceptor units"
by Haiyan Tian, Xi Tang and Yong Qiang Dong

Corresponding author:
Beijing Key Laboratory of Energy Conversion and Storage Materials, Department of Chemistry, Beijing Normal University, Beijing, China;
E-mail: dongyq@bnu.edu.cn

Table S1 Crystal data and structure refinement for the single crystal of **1GC**

Empirical formula	$C_{40}H_{24}N_4$	
Formula weight	560.63	
Temperature	100(2) K	
Wavelength	0.71073 A	
Crystal system, space group	Orthorhombic, P2(1)2(1)2(1)	
	a = 7.907(2) A	alpha = 90 deg.
Unit cell dimensions	b = 14.819(4) A	beta = 90 deg.
	c = 26.414(7) A	gamma = 90 deg.
Volume	3095.1(13) A^3	
Z, Calculated density	4, 1.203 Mg/m^3	
Absorption coefficient	0.072 mm^-1	
F(000)	1168	
Crystal size	0.410 x 0.200 x 0.110 mm	
Theta range for data collection	2.066 to 25.250 deg.	
Limiting indices	-9<=h<=9, -17<=k<=17, -31<=l<=29	
Reflections collected / unique	17606 / 5609 [R(int) = 0.0522]	
Completeness to theta = 25.242	99.90%	
Absorption correction	Semi-empirical from equivalents	
Max. and min. transmission	0.75 and 0.64	
Refinement method	Full-matrix least-squares on F^2	
Data / restraints / parameters	5609 / 0 / 397	
Goodness-of-fit on F^2	1.031	
Final R indices [I>2sigma(I)]	R1 = 0.0525, wR2 = 0.1259	
R indices (all data)	R1 = 0.0717, wR2 = 0.1387	
Absolute structure parameter	0.1(10)	
Extinction coefficient	n/a	
Largest diff. peak and hole	0.891 and -0.204 e.A^-3	

Table S2 Crystal data and structure refinement for the single crystal of **1YC**

Empirical formula	C_{44} H_{32} N_{4} O	
Formula weight	632.73	
Temperature	100(2) K	
Wavelength	0.71073 A	
Crystal system, space group	Monoclinic, P2(1)	
	a = 10.3034(16) A	alpha = 90 deg.
Unit cell dimensions	b = 23.763(4) A	beta = 104.447(3) deg.
	c = 13.737(2) A	gamma = 90 deg.
Volume	3257.0(8) A^3	
Z, Calculated density	4, 1.290 Mg/m^3	
Absorption coefficient	0.078 mm^-1	
F(000)	1328	
Crystal size	0.400 x 0.370 x 0.300 mm	
Theta range for data collection	1.714 to 25.249 deg.	
Limiting indices	-12<=h<=6, -27<=k<=28, -16<=l<=16	
Reflections collected / unique	18602 / 11381 [R(int) = 0.0358]	
Completeness to theta = 25.242	99.90%	
Absorption correction	Semi-empirical from equivalents	
Max. and min. transmission	0.75 and 0.65	
Refinement method	Full-matrix least-squares on F^2	
Data / restraints / parameters	11381 / 1 / 883	
Goodness-of-fit on F^2	1.045	
Final R indices [I>2sigma(I)]	R1 = 0.0533, wR2 = 0.1270	
R indices (all data)	R1 = 0.0650, wR2 = 0.1358	
Absolute structure parameter	-1.1(10)	
Extinction coefficient	n/a	
Largest diff. peak and hole	0.415 and -0.280 e.A^-3	

Table S3 Crystal data and structure refinement for the single crystal of **1OC**

Empirical formula	C40 H24 N4
Formula weight	560.63
Temperature	100(2) K
Wavelength	0.71073 A
Crystal system, space group	Orthorhombic, Pbcn
	a = 22.459(5) A alpha = 90 deg.
Unit cell dimensions	b = 16.839(4) A beta = 90 deg.
	c = 7.8968(18) A gamma = 90 deg.
Volume	2986.5(12) A^3
Z, Calculated density	4, 1.247 Mg/m^3
Absorption coefficient	0.074 mm^-1
F(000)	1168
Crystal size	0.350 x 0.340 x 0.100 mm
Theta range for data collection	1.813 to 27.522 deg.
Limiting indices	-29<=h<=20, -21<=k<=21, -10<=l<=10
Reflections collected / unique	18984 / 3436 [R(int) = 0.0441]
Completeness to theta = 25.242	100.00%
Absorption correction	Semi-empirical from equivalents
Max. and min. transmission	0.75 and 0.66
Refinement method	Full-matrix least-squares on F^2
Data / restraints / parameters	3436 / 0 / 200
Goodness-of-fit on F^2	1.037
Final R indices [I>2sigma(I)]	R1 = 0.0407, wR2 = 0.0875
R indices (all data)	R1 = 0.0570, wR2 = 0.0958
Extinction coefficient	n/a
Largest diff. peak and hole	0.261 and -0.217 e.A^-3

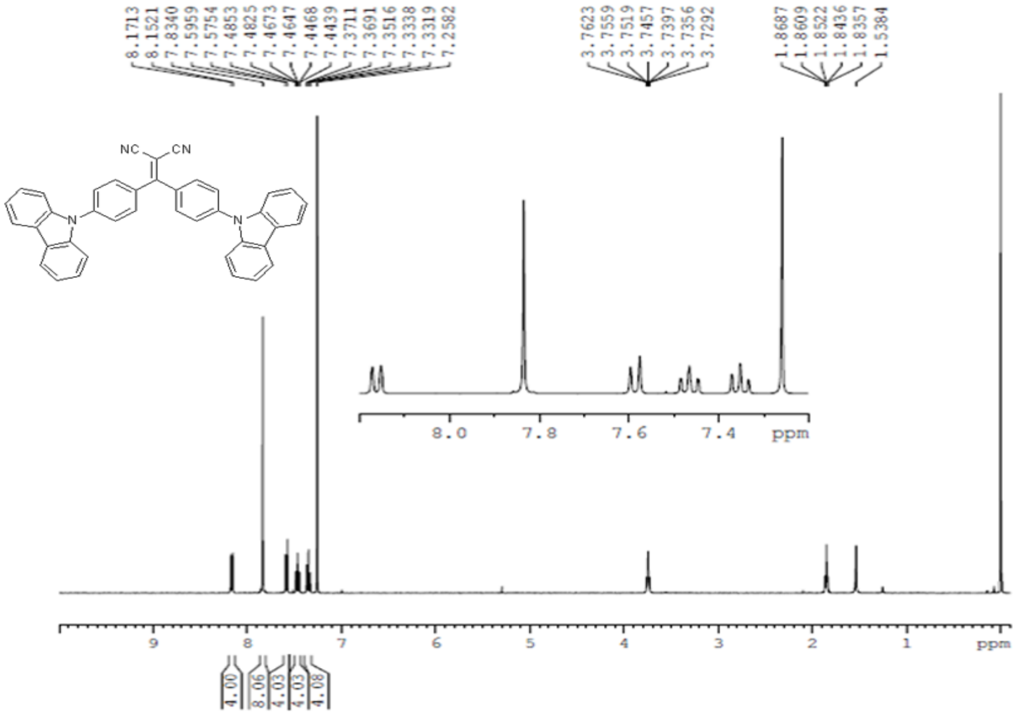

Figure S1. The ¹H NMR spectrum of **1YC** in $CDCl_3$ solvent.

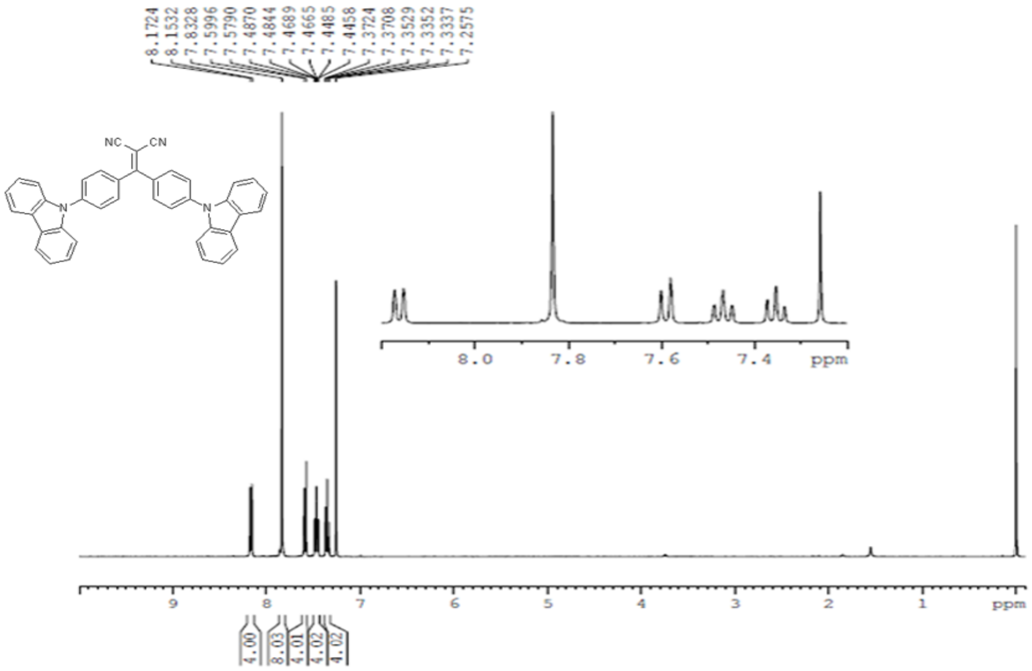

Figure S2. The ¹H NMR spectrum of **1YC** after heating at 90°C in vacuum in $CDCl_3$ solvent.

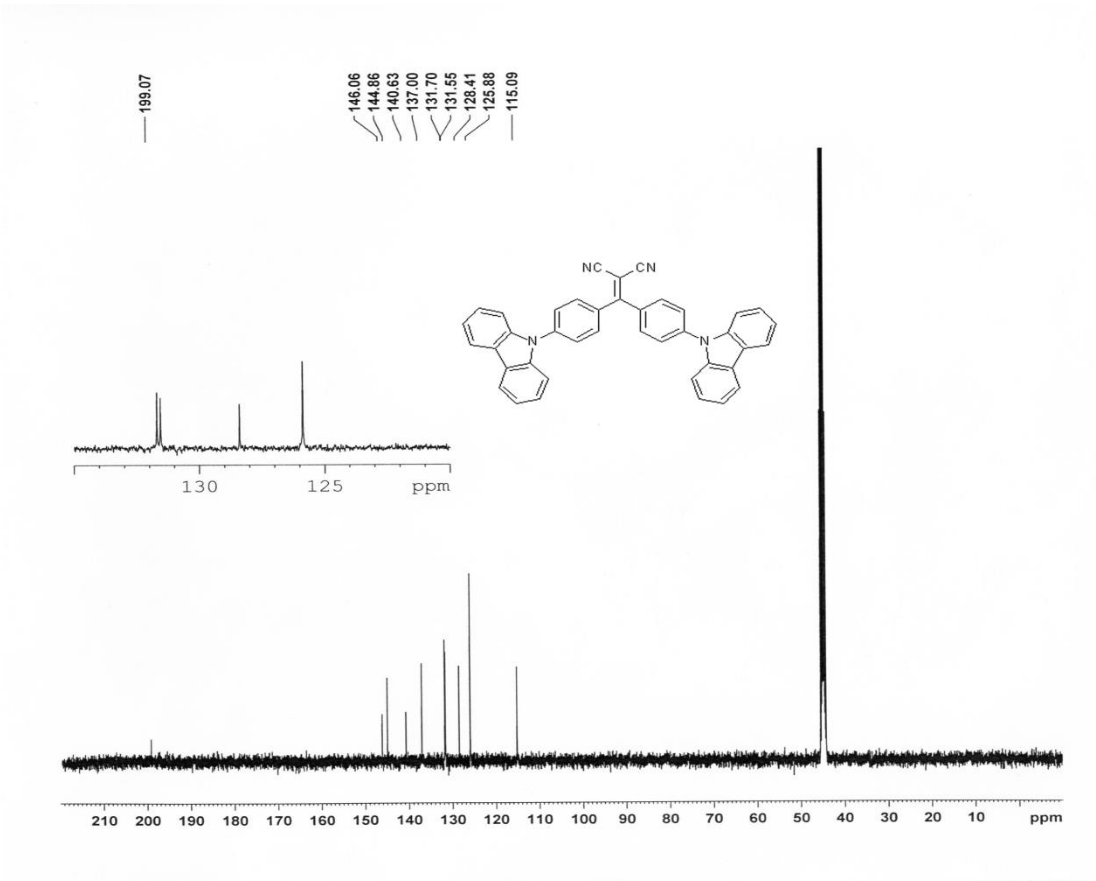

Figure S3. The ^{13}C NMR spectrum of **1** in DMSO solvent.

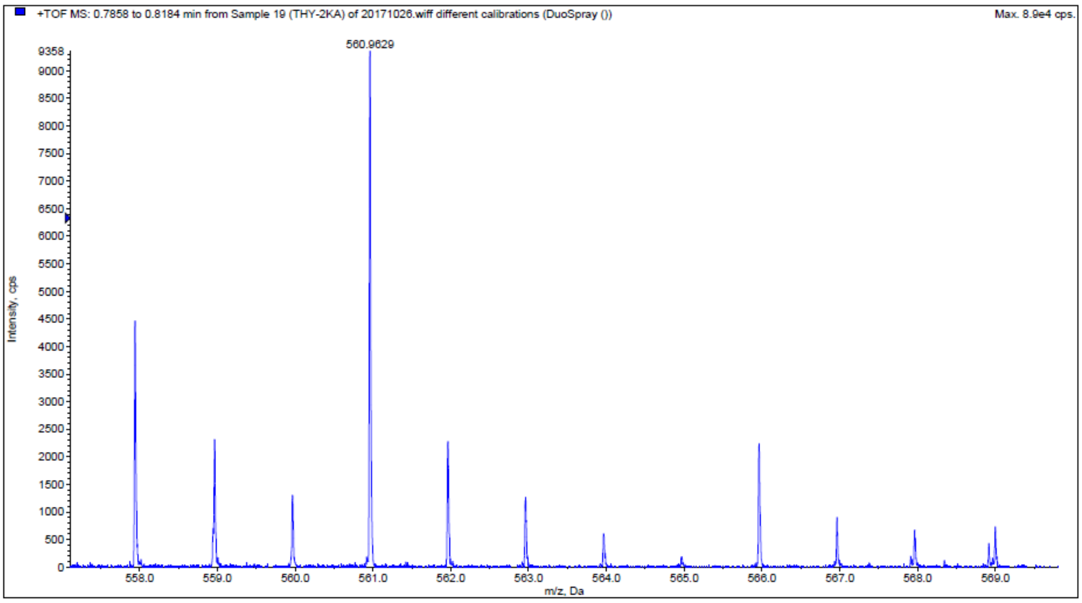

Figure S4. The HRMS spectrum of compound **1.**

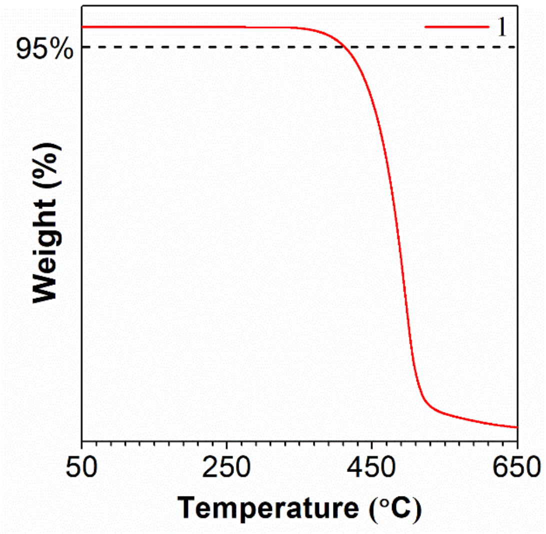

Figure S5. TGA thermograms of the **1** recorded under nitrogen at a heating rate of 10 K/min.

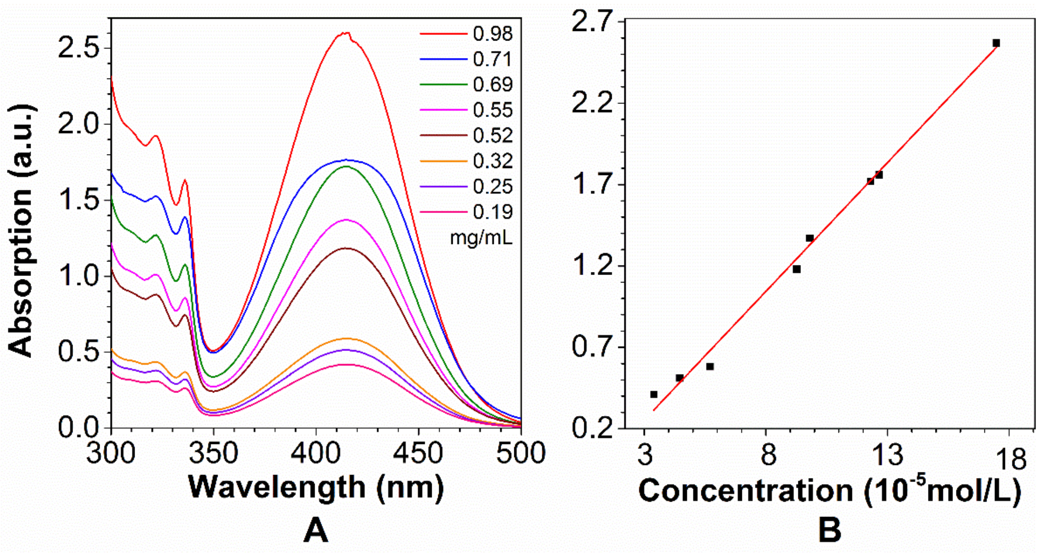

Figure S6. (**A**)The UV-vis absorption spectra of **1** in DCM versus different concentration (mg/mL). (**B**) Linear fitting about absorption intensity versus concentration (mol/L), $R^2 = 0.992$.

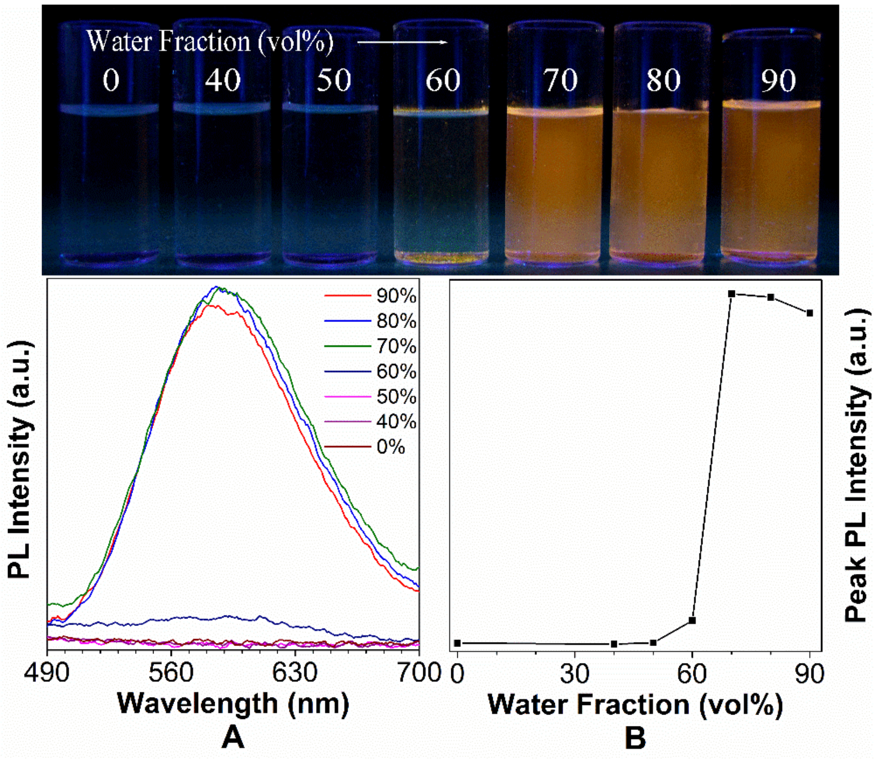

Figure S7. Photographs taken under 365nm UV light illumination. (**A**) PL spectra of **1** in acetonitrile/water mixtures with different water fractions (f_w, vol %). (**B**) Plots of maximum emission intensity versus water fractions. Concentration: 1 μM; excitation wavelength: 370 nm; exposure time: 2 second.

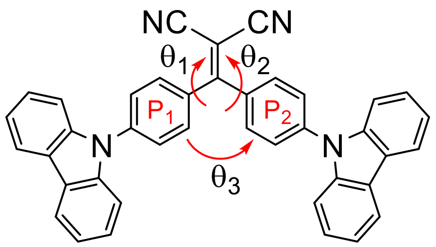

Table S4 Torsion angle of phenyl rings in three single crystals of compound **1.**

Samples	λ_{em} (nm)	θ_1 (°)	θ_2 (°)	θ_3 (°)
**1GC**	506	41.99	50.36	73.85
**1YC**	537	34.35	46.21	67.79
**1OC**	585	43.03	43.03	73.27

Dihedral angle of **1** in different crystals. θ_1, dihedral angle between benzene ring plane P_1 and double bond plane; θ_2, dihedral angle between benzene ring plane P_2 and double bond plane; θ_3, dihedral angle of plane P_1 and P_2.

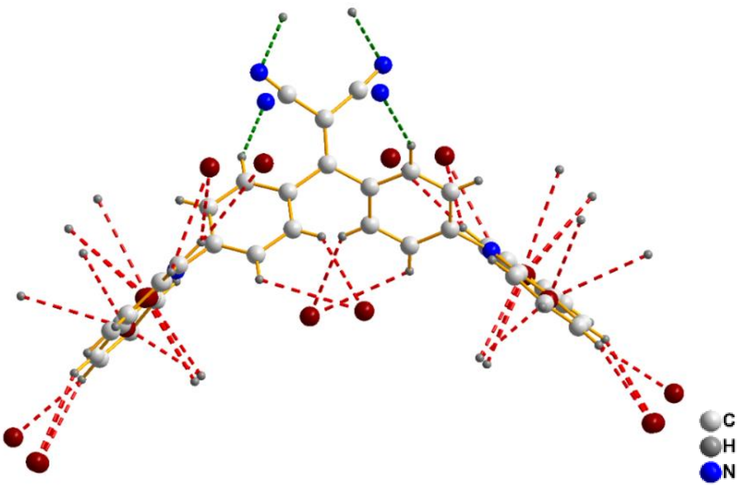

Figure S8. View of C≡N···H (green dashed line) and C-H···π (red dashed line) intermolecular interactions in single crystal of **1OC**. The dark-red dots refer to the center of benzene rings.

Table S5 Summarization of the C≡N···H and C-H···π Interactions in the Crystal of **1OC**.

Interactions	d /Å [a](N)[b]	A/° [c]
1C≡N···H	2.673(4)	155.016
2C-H···π	2.726(4)	159.282
3C-H···π	3.042(4)	125.192
4C-H···π	3.058(4)	138.157
5C-H···π	3.106(4)	122.975
6C-H···π	3.119(4)	156.444
7C-H···π	3.197(4)	160.526
8C-H···π	3.374(4)	124.254
9C-H···π	3.390(4)	122.774
10C-H···π	3.439(4)	120.326

[a] Distance of C≡N···H or C-H···π interaction. [b] Number of the intermolecular interactions. [c] Angel of C≡N···H or C-H···π interaction.

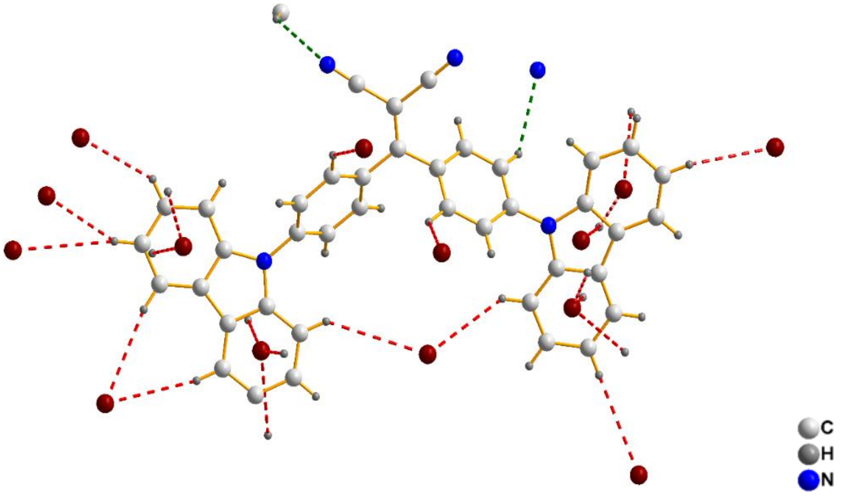

Figure S9. View of C≡N···H (green dashed line) and C-H···π (red dashed line) intermolecular interactions in single crystal of **1GC**. The dark-red dots refer to the center of benzene rings.

Table S6 Summarization of the C≡N···H and C-H···π Intermolecular Interactions in the Crystal of **1GC.**

Interactions	d /Å [a](N)[b]	A/° [c]
1C≡N···H	2.667(2)	123.372
2C-H···π	2.745(2)	139.18
3C-H···π	2.774(2)	152.432
4C-H···π	2.787(2)	143.257
5C-H···π	3.079(2)	128.045
6C-H···π	3.130(2)	137.635
7C-H···π	3.173(2)	169.382
8C-H···π	3.179(2)	169.559
9C-H···π	3.232(2)	148.758
10C-H···π	3.359(2)	163.27
11C-H···π	3.394(2)	167.583
12C-H···π	3.548(2)	140.914

[a] Distance of C≡N···H or C-H···π interaction. [b] Number of the intermolecular interactions. [c] Angel of C≡N···H or C-H···π interaction.

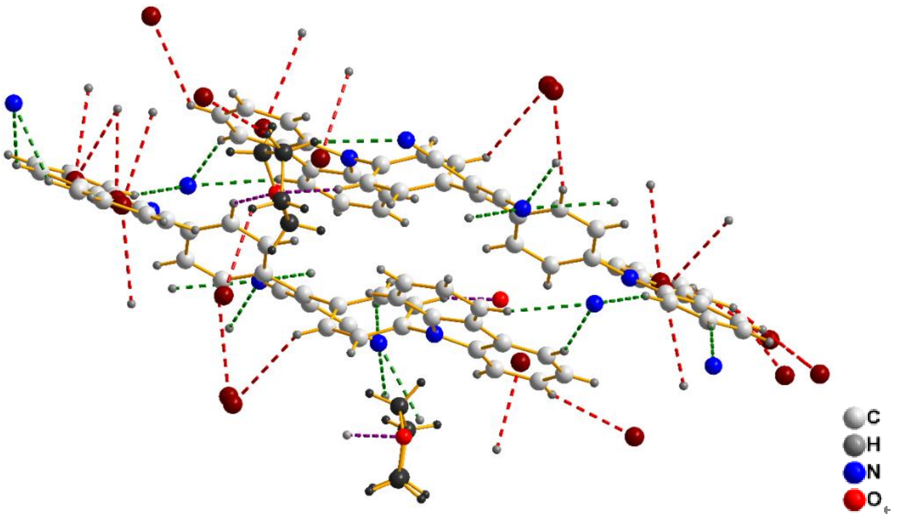

Figure S10. View of C-H···O (purple dashed line), C≡N···H (green dashed line) and C-H···π (red dashed line) intermolecular interactions in single crystal of **1YC**. The dark-red dots refer to the center of benzene rings.

Table S7 Summarization of the C-H···O, C≡N···H and C-H···π Intermolecular Interactions in the crystal of **1YC**.

Interactions	d /Å [a](N)[b]	A/° [c]
1C-H···O	2.573(2)	139.779
2C-H···O	2.660	141.589
3C-H···O	2.612	145.501
4C≡N···H	2.719(2)	164.219
5C≡N···H	2.790(2)	149.293
6C≡N···H	2.546(2)	155.263
7C≡N···H	2.739(2)	158.635
8C≡N···H	2.694(2)	157.567
9C≡N···H	2.667(2)	153.592
10C≡N···H	2.666(2)	152.324
11C≡N···H	2.800	161.529
12C≡N···H	2.691(2)	127.869
13C≡N···H	2.699(2)	127.791
14C-H···π	3.020(3)	132.551
15C-H···π	2.826(2)	142.937
16C-H···π	3.404(2)	154.303
17C-H···π	2.858(2)	163.874
18C-H···π	3.148(2)	145.282
19C-H···π	3.130(2)	149.187
20C-H···π	3.143(3)	128.328
21C-H···π	3.524(2)	124.632
22C-H···π	2.765(2)	165.666
23C-H···π	3.187(2)	144.7355

[a] Distance of C-H···O, C≡N···H or C-H···π interaction. [b] Number of the intermolecular interactions. [c] Angel of C-H···O, C≡N···H or C-H···π interaction.

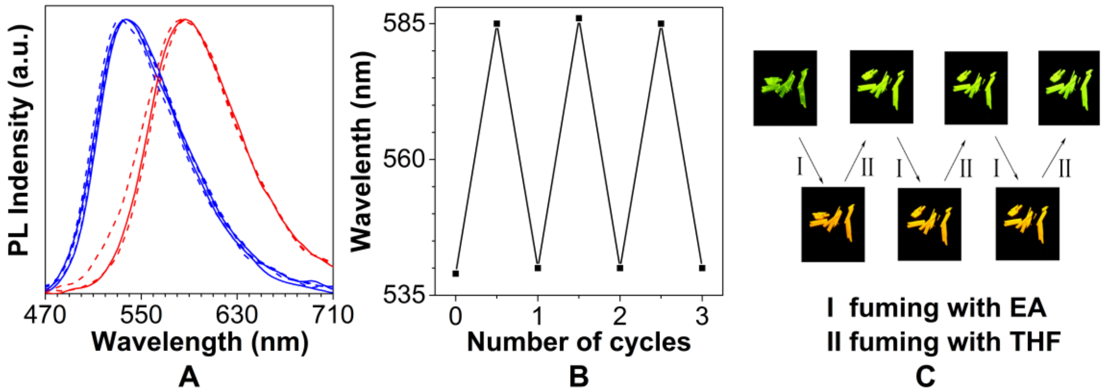

Figure S11. (**A**) Normalized PL spectra of fumed solid of **1** in the three repeating cycles; excitation wavelength: 370 nm. (**B**) Switching the fluorescence of **1** by repeated fuming with EA (Ⅰ) and THF (Ⅱ) on the quartz plate. (**C**) Digital photograph three repeating cycles under illuminant of 365 nm.

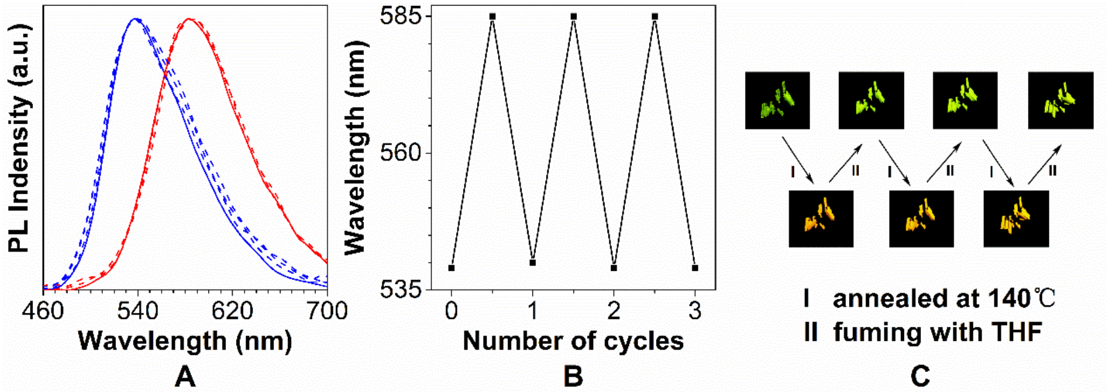

Figure S12. (**A**) Normalized PL spectra of heated and fumed solid of **1** in the three repeating cycles. Excitation wavelength: 370 nm. (**B**) Switching the fluorescence of **1** by repeated annealing at 140℃, (Ⅰ) and fuming with THF, (Ⅱ) on the quartz plate. (**C**) Digital photograph of the three repeating cycles under illuminant of 365 nm.

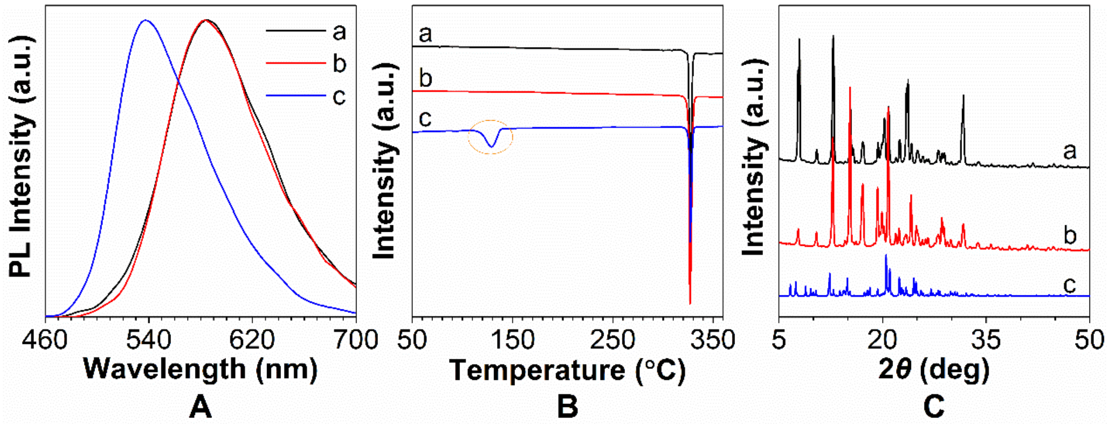

Figure S13. (**A**) Normalized PL spectra, (**B**) DSC curves and (**C**) PXRD patterns of **1** in the first repeating cycle: (a) **1OC**, (b) **1YC** annealed at 140℃, (c) **1YC**.
